# Finding directionality and gene-disease predictions in disease associations

**DOI:** 10.1186/s12918-015-0184-9

**Published:** 2015-07-15

**Authors:** Manuel Garcia-Albornoz, Jens Nielsen

**Affiliations:** Department of Biology and Biological Engineering, Chalmers University of Technology, Göteborg, Sweden

## Abstract

**Background:**

Understanding the underlying molecular mechanisms in human diseases is important for diagnosis and treatment of complex conditions and has traditionally been done by establishing associations between disorder-genes and their associated diseases. This kind of network analysis usually includes only the interaction of molecular components and shared genes. The present study offers a network and association analysis under a bioinformatics frame involving the integration of HUGO Gene Nomenclature Committee approved gene symbols, KEGG metabolic pathways and ICD-10-CM codes for the analysis of human diseases based on the level of inclusion and hypergeometric enrichment between genes and metabolic pathways shared by the different human disorders.

**Methods:**

The present study offers the integration of HGNC approved gene symbols, KEGG metabolic pathways andICD-10-CM codes for the analysis of associations based on the level of inclusion and hypergeometricenrichment between genes and metabolic pathways shared by different diseases.

**Results:**

880 unique ICD-10-CM codes were mapped to the 4315 OMIM phenotypes and 3083 genes with phenotype-causing mutation. From this, a total of 705 ICD-10-CM codes were linked to 1587 genes with phenotype-causing mutations and 801 KEGG pathways creating a tripartite network composed by 15,455 code-gene-pathway interactions. These associations were further used for an inclusion analysis between diseases along with gene-disease predictions based on a hypergeometric enrichment methodology.

**Conclusions:**

The results demonstrate that even though a large number of genes and metabolic pathways are shared between diseases of the same categories, inclusion levels between these genes and pathways are directional and independent of the disease classification. However, the gene-disease-pathway associations can be used for prediction of new gene-disease interactions that will be useful in drug discovery and therapeutic applications.

**Electronic supplementary material:**

The online version of this article (doi:10.1186/s12918-015-0184-9) contains supplementary material, which is available to authorized users.

## Background

In medical research the use of computational and mathematical tools for analysing large networks between genes, diseases and metabolic pathways has gained increasing interest in recent years [1–7]. This analysis has led to the discovery of associations between phenotypes and disease genes, enabling the discovery of comorbidities and disease associations providing potentially important tools for disease diagnosis and prevention [8]. Comorbidity has an impact on the diagnosis, choice of treatment, morphology and rate of survival in patients with different diseases such as cancer.

In order to understand the associations that lead to an observed phenotype in human diseases, several interactions have been explored and several strategies have been developed in order to analyse the information contained in these disease network. It has been stated that disease modules are highly interconnected considering that perturbations caused by one disease can affect other diseases, and a diseasome has been coined to systematically map such network-based relationships between diseases where nodes are diseases and edges represent different studies showing comorbidities. Among the molecular relationships linking disease associations in network analysis are genes, metabolic pathways, microRNA and phenotypes [1, 8–10]. However, despite the discovery of shared biological roles between highly connected nodes, analysing the level and directionality of inclusion between genes and metabolic pathways shared by different diseases can lead to the formulation of new hypotheses based on the directionality of the associations. We therefore generated a network of genes, metabolic pathways and diseases, and in order to avoid difficulties with changes in gene names and disease classifications we used standardised nomenclature for diseases, genes and pathways. This network was further adapted and used in the development of an inclusion study between diseases and in an enrichment analysis aiming for the discovery of new disease-related genes.

## Results and discussions

### Disease network

We integrated the HUGO Gene Nomenclature Committee (HGNC) approved gene symbols and ICD-10-CM (International Classification of Diseases) codes for diseases classification in order to provide the most updated human disease network to date. The disease-gene associations were obtained from the OMIM database that offers an updated list of phenotypes for which the molecular basis is known in association of genes with phenotype-causing mutation. As of the time of the study, the downloaded OMIM morbidity map included a collection of 4315 phenotypes and 3083 genes with phenotype-causing mutation. The original OMIM morbid map included a list of 9102 genes with phenotype-causing mutation producing 15,310 gene-disease interactions, but when the database was updated following HGNC rules for approved gene symbols the list was reduced to 3083 genes and 4618 interactions. We believe it is important to incorporate the HGNC rules as genes are constantly being reviewed and updated including name and symbol changes or locus type reclassification by the HGNC, which is the only worldwide authority responsible for assignation of standardised symbols to human genes [11].

**Fig. 1:**
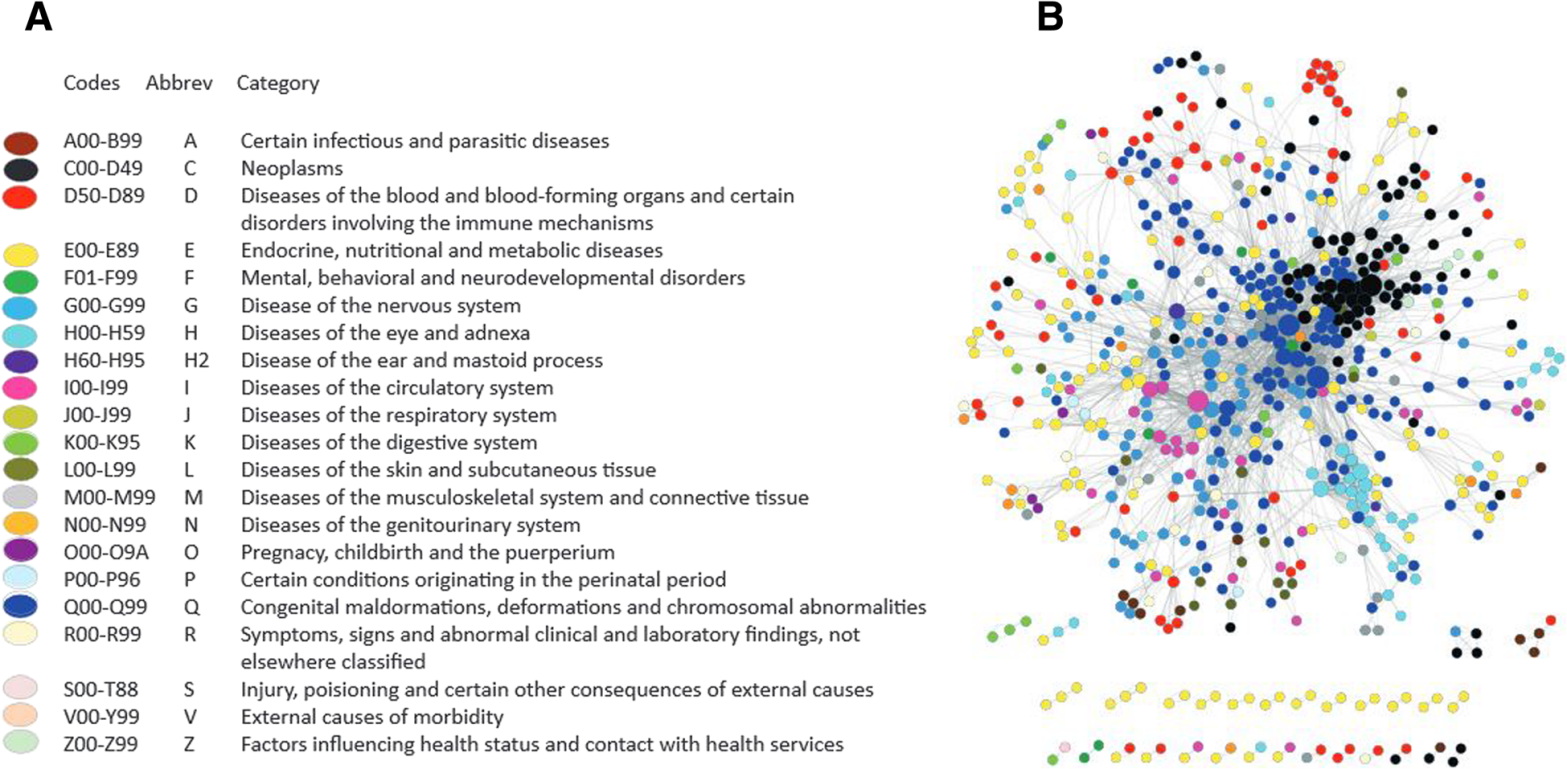
Disease-disease interactions. **a** ICD-10-CM code classification. **b** Bipartite disease-disease network for 4315 OMIM phenotypes classified into 880 unique ICD-10-CM codes. Nodes represent codes and edges represent shared genes. The size of the node denotes the number of edges involved in each code

The established database was manually curated in order to assign the correspondent ICD-10-CM code to each of the OMIM phenotypes. The ICD-10-CM is the 10th revision of the medical classification list by the World Health Organization (WHO). It is intended to be the standard diagnostic tool for epidemiology, health management and clinical purposes and depending on the country the ICD-10-CM codes are used for reimbursement and resource allocation decision-making. The classification includes more than 14,000 different codes allowing expansion to over 16,000 by using optional sub-classifications. The ICD-10-CM codes are organized in twenty one main categories (Fig. 1a) from which several sub-categories are developed until specific codes are assigned to each disease.

**Fig. 2:**
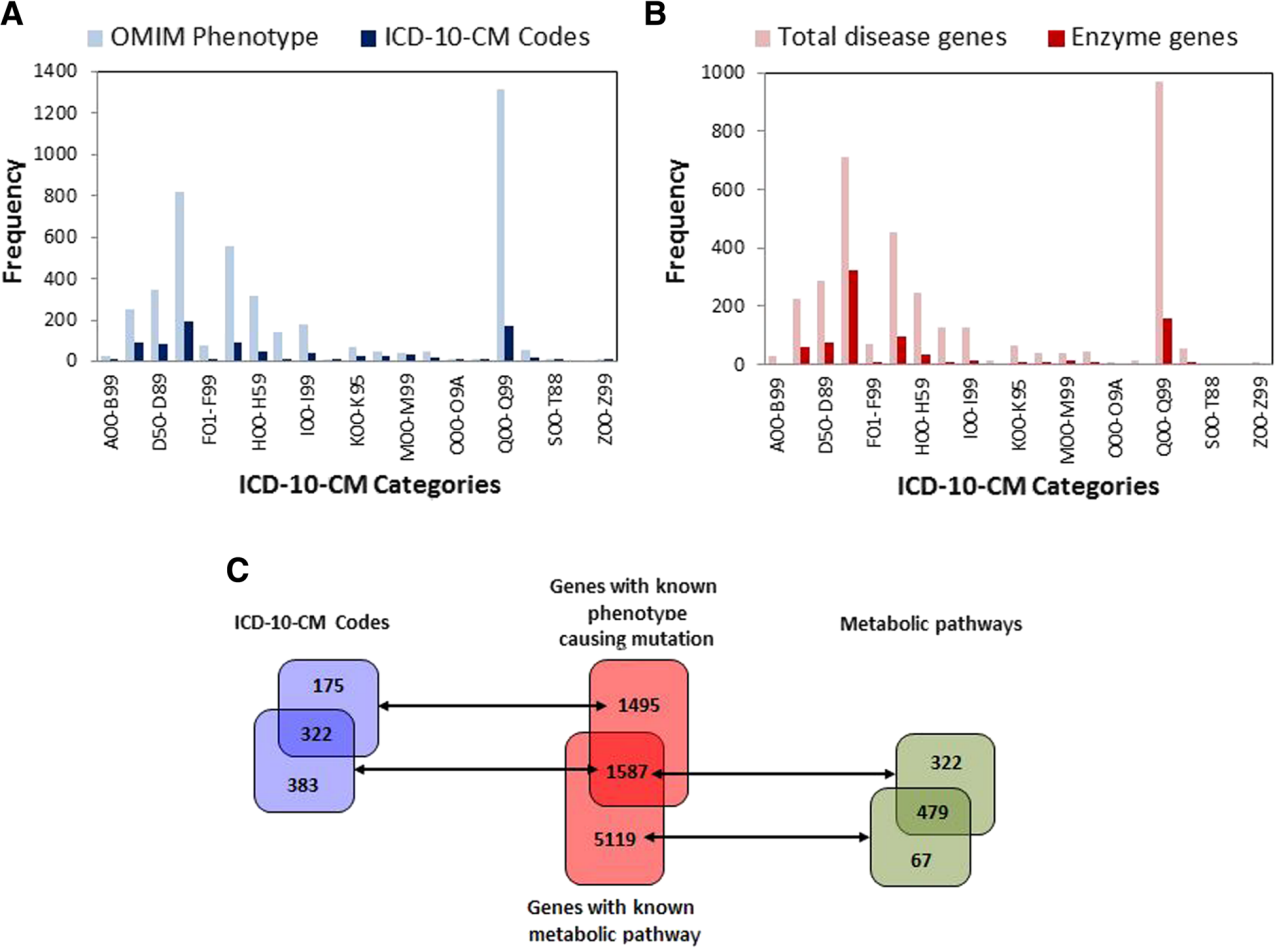
Gene and disease network analysis. **a** Number of OMIM phenotypes and codes by ICD-10-CM category. **b** Number of unique disease-genes by ICD-10-CM category and the number of enzyme-producing genes. **c** 880 unique ICD-10-CM codes are linked to 3083 genes with phenotype-causing mutation creating 4241 disease-gene associations. Of the 880 codes a total of 705 codes and 1587 genes are linked to 801 metabolic pathways creating 15,455 code-gene-pathway interactions. A further analysis revealed a total of 6706 genes being involved in at least one KEGG metabolic pathway, and hereby 5119 genes with no known phenotype-causing mutation could be included in our analysis. These 5119 genes with no known phenotype-causing mutation are linked to 546 different KEGG pathways sharing 479 pathways with genes carrying phenotype-causing mutation and are only linked to 67 additional KEGG metabolic pathways

880 unique ICD-10-CM codes were mapped to the 4315 OMIM phenotypes in the database, and a bipartite network was developed linking the 880 unique codes with the 3083 genes carrying phenotype-causing mutations resulting in 4241 disease-gene associations. Based on this bipartite network, 3430 interactions (disease-pairs) were found between diseases sharing at least one gene. This network is showed in Fig. 1b in which each node is an ICD-10-CM code and the edges are gene-disease association linking two codes if they share at least one gene. The size of the node denotes the number of genes (edges) involved in the disease and the width of the edge is proportional to the number of genes shared by both diseases. The main advantage of the ICD-10-CM classification is the grouping of some of the OMIM diseases into a particular code creating disease associations based on shared disease names which can be traced hierarchically to obtain larger disease clusters by main categories. Figure 1b shows some of the clusters formed in the disease network for different disease classifications such as neoplasms (C00-D49), congenital malformations (Q00-Q99), disease of the eye (H00-H59), diseases of the circulatory system (I00-I99) and diseases of the blood (D50-D89). Hereby it is possible to easily explore the level of association between diseases corresponding to the same classification. Due to the characteristics of the OMIM phenotypes with validated gene-disease associations, it is anticipated that some of the ICD-10-CM codes have no link with any OMIM phenotype. This is the case for external causes of morbidity (V00-Y99) and injury, poisoning and certain other consequences of external causes (S00-T88) (Fig. 2a).

Figure 2b shows the number of unique genes involved in each corresponding disease classification group along with the number of enzyme-encoding genes. It is noticeable that 717 of the 3083 disease-genes are enzymes, covering 25 % of the enzymes reported in the HPA database [12]. In order to evaluate whether there is any enrichment of specific metabolic pathways associated with specific diseases, we added to the gene-disease associations a link to metabolic pathways, and hereby created associations between metabolic pathways, genes and diseases. With this, we expected to expand the possibility of disease associations by establishing more complex mechanism underlying the disease-disease networks. For this purpose, a tripartite network was created linking diseases and genes with their associated metabolic pathways from the KEGG database. For the study, pathways (ko), modules (M) and diseases (H) from the KEGG database were included in order to increase the level of interaction between diseases. The 3083 disease-genes were mapped to the KEGG database from where a total of 1587 genes were linked to 801 KEGG pathways; these genes were mapped with 705 ICD-10-CM codes creating a tripartite network composed by 15,455 code-gene-pathway interactions (Fig. 2c). From this tripartite network 112,956 associations (disease-pairs) were found between diseases sharing at least one pathway.

A further analysis was made to create links between genes with no known phenotype-causing mutation and pathways based on KEGG database. The whole HGNC gene database was then mapped to the KEGG database finding a total of 6706 genes being involved in at least one KEGG metabolic pathway. 5119 genes with no known phenotype-causing mutation were linked to 546 different KEGG pathways; sharing 479 pathways with genes carrying phenotype-causing mutation (see Fig. 2c).

### Inclusion analysis

**Fig. 3:**
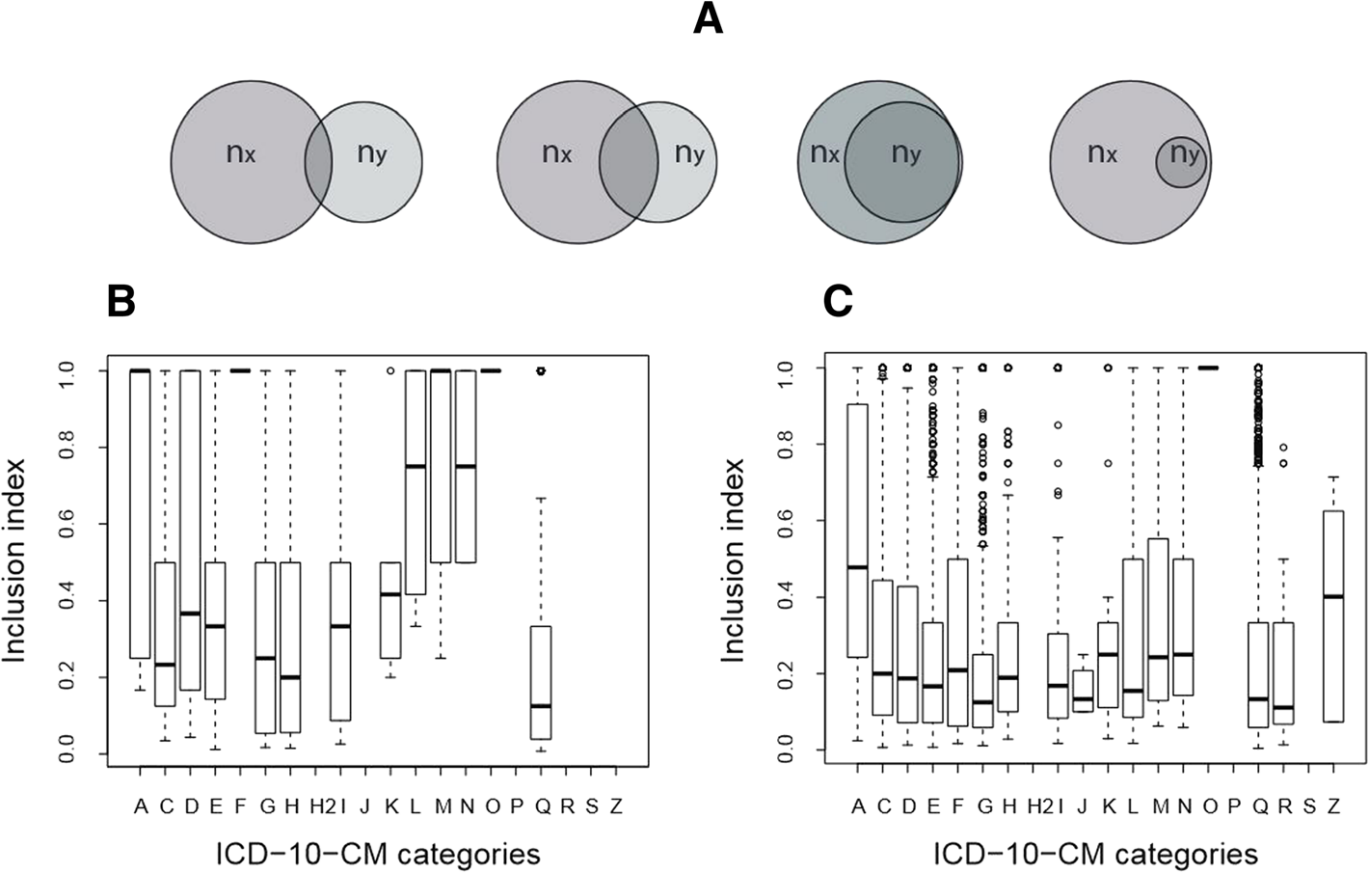
Inclusion analysis by disease category. **a** For two diseases sharing a certain number of elements (genes or pathways), the inclusion index (*τ*) will be low for a disease with a high number of total elements compared with the number of shared elements. When the number of shared elements increases compared with the total number of elements of the disease, the index level increases. Therefore, different index values can be calculated for two diseases sharing elements depending on the total number of elements of each disease. Consequently, this index allows obtaining not only the degree of interaction between diseases, but also the directionality of the interaction. A value of *τ* = 1 indicates that one disease is a subset of another. **b** Boxplot of calculated level of inclusion between disease-pairs belonging to the same ICD-10-CM category based on shared genes. **c** Boxplot of calculated level of inclusion between disease-pairs belonging to the same ICD-10-CM category based on shared pathways

 In order to obtain a more robust analysis of the disease-disease associations, the level of inclusion was calculated for all the 3430 disease-disease pairs sharing at least one gene and for all the 112,956 disease-disease pairs sharing at least one pathway. This information is important since the number of shared genes or pathways between diseases compared to the total pool is different for each disease, therefore, the inclusion index (*τ*) allows establishing not only the number of genes or pathways shared between two diseases but at what degree the genes or pathways of disease *X* are contained in disease *Y* which can lead to the discovery of subsets of elements between diseases (Fig. 3a). The study reveals that the level of inclusion based on shared genes between disease-pairs belonging to the same ICD-10-CM category (Fig. 3b) is high for 6 disease categories: infectious and parasitic diseases (A00-B99), mental, behavioural and neurodevelopmental disorders (F01-F99), diseases of the skin and subcutaneous tissue (L00-L99), diseases of the musculoskeletal system and connective tissue (M00-M99), diseases of the genitourinary system (N00-N99) and diseases during pregnancy, childbirth and the puerperium (O00-O9A). When the study is done by shared pathways (Fig. 3c), the diseases show poor level of inclusion inside their category with the exception of pregnancy and childbirth diseases (O00-O9A).

It is important to notice the existence of certain disease categories where there is no inclusion between genes or pathways. This can be as a result of the current characteristics of the human-disease classification which is based on clinical features and do not take into account the underlying molecular basis shared by a group of diseases. This is emphasized by the fact that in the ICD-10 classification it is possible for certain diseases to be classified into two different groups, showing a lack of understanding of the real disease mechanism. The results are important to address if the official disease classification is helpful in order to give the right diagnosis and treatment of human conditions and going further, the lack of understanding of the molecular mechanism under the development of human diseases can poorly explain the occurrence of comorbidities and their development. Adding to this, some diseases are so phenotypically complicated that the ICD classification relies on general classifications such as “Other malformations not elsewhere classified” which includes a large amount of non-understood human conditions and newly found perturbations.

**Fig. 4:**
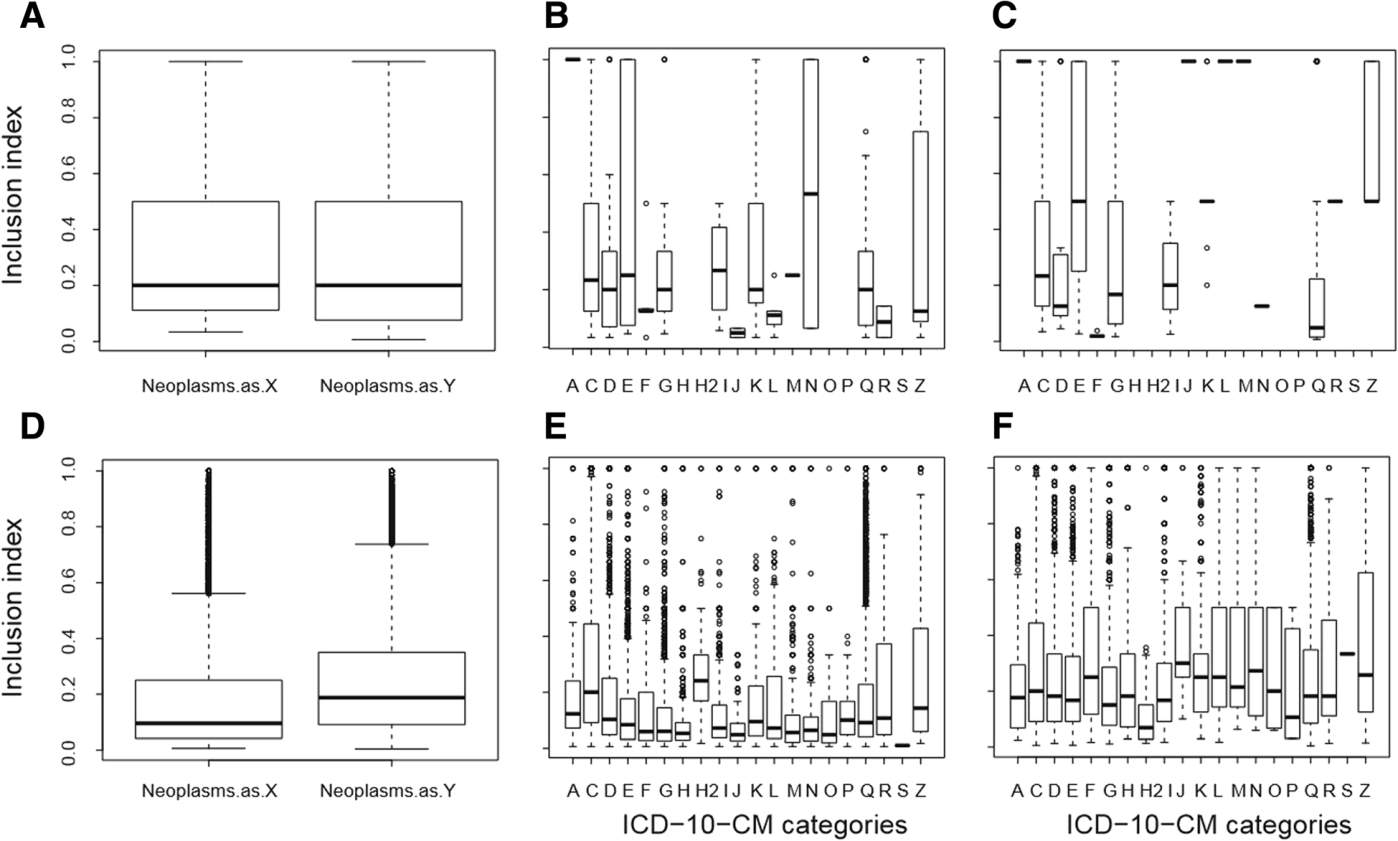
Inclusion analysis for Neoplasm category. **a** Boxplot of calculated level of inclusion (*τ*) based on shared genes for neoplasms as disease X and Y. **b** Boxplot of calculated level of inclusion based on shared genes for neoplasms as disease X by ICD-10-CM category. **c** Boxplot of calculated level of inclusion based on shared genes for neoplasms as disease Y by ICD-10-CM category. **d** Boxplot of calculated level of inclusion based on shared pathways for neoplasms as disease X and Y. **e** Boxplot of calculated level of inclusion based on shared pathways for neoplasms as disease X by ICD-10-CM category. **f** Boxplot of calculated level of inclusion based on shared pathways for neoplasms as disease Y by ICD-10-CM category

Somewhat surprising the neoplasm category was showing low levels of inclusion based on both genes and pathways. A detailed study was done in order to understand their level of interaction. In Fig. 4a it can be seen that neoplasms have no significant difference in the directionality of inclusion when act like either disease *X* or disease *Y*, meaning that they show a general low level of inclusion when being included by or including genes of other diseases. However, a detailed study by ICD-10-CM classification shows that this directionality is different between ICD-10-CM categories with some categories showing low level of inclusion of genes in neoplasms but being totally included by the neoplasms (Fig. 4b and c). Genes in diseases of the respiratory system (J00-J99), diseases of the skin and subcutaneous tissue (L00-L99) and diseases of the musculoskeletal system and connective tissue (M00-M99) have a big change in directionality and happens to be subsets of certain neoplasms, but not in the other way round with the exception of certain infectious and parasitic disease (A00-B99) showing total inclusion in both directions. When the analysis is made by shared pathways the results show a small increase in the level of inclusion when neoplasms change from disease *X* to disease *Y*, meaning that they are more prone to include pathways of other diseases rather than being included by these diseases. However, results show long tails and deviations, meaning that this behaviour is not generalized and every neoplasm should be analysed independently and not as part of a general classification (Fig. 4d, e and f). Since neoplasms show a general tendency to include pathways and genes from other diseases rather than being included, this can give a hint about the comorbidities observed in cancer patients. In a recent publication, it was shown that, according to different studies, comorbidity is common in patients with colon cancer (14 %–68 %), breast cancer (20 %–35 %) and lung cancer (26 %–81 %) with the percentage of occurrence depending on the demographic characteristics of the study. In this studies, it was found that for these three types of cancer, diabetes, chronic obstructive pulmonary disease, congestive heart failure and cerebrovascular disease are among the most common comorbidities [13].

### Gene-disease prediction

The disease-gene-pathway associations created in the present study were further explored in order to evaluate the usefulness of the network for gene-disease predictions. As mentioned before, 5119 genes with no known phenotype-causing mutation were linked to 546 different KEGG pathways. This 5119 genes share 479 pathways with genes having phenotype-causing mutation, and we can hereby predict new putative genes involved in different diseases not yet reported in the OMIM database. A total of 348,882 disease-gene pairs were found sharing at least one pathway and the statistical significance of the shared metabolic pathways was calculated for each pair using a two-tailed Fisher’s test. From the total of 348,882 disease-gene pairs evaluated using the standard hypergeometric distribution, 31,066 pairs were enriched with a p-value ≤ 0.001. Table 1 shows the top 10 rated gene-disease pairs. We demonstrated the ability of our network-enrichment method to produce relevant results by finding genes that are being already analysed as potential targets in different gene-disease associations. Although we demonstrated the utility of our method on the KEGG pathways, it is equally applicable to other classifications or enrichment methods. Among the top rated gene-disease pairs are genes previously reported/suggested as candidates in different diseases. This consistency with previously reported clinical findings not included in the OMIM database demonstrates the predictive power of the methodology. The analysis produced three top genes related to several cancer types, *PIK3CB*, *PIK3CG* and *PIK3R3* that have been previously reported to be involved in several cancer types with bidirectional gene expression. *PIK3CB* has been studied in breast cancer [14], and in PTEN-deficient cancers [15]. *PIK3CG* has been reported as a candidate myeloid tumour suppressor [16], and as candidate in the growth and progression of colorectal cancers [17]. *PIK3R3* expression and function has been studied in Asian patients with gastric cancer [18], and in metastasis promotion in colorectal cancer [19].

**Table 1:** Top 10 rated gene-disease pairs

Gene	Disease
*PIK3CB*	Neoplasms, seborrheic keratosis
*PIK3CG*	Neoplasms, seborrheic keratosis
*PIK3R3*	Neoplasms, seborrheic keratosis
*SOS2*	Gingival enlargement
*GRB2*	Gingival enlargement
*MAPK1*	Neoplasms
*MAPK3*	Neoplasms
*PLCB3*	Epilepsy
*RELA*	Streptococcal infection
*NFKB1*	Streptococcal infection, immunodeficiency

Top 10 rated gene-disease pairs using the hypergeometric distribution

These three genes have been as well top rated for their role in seborrheic keratosis, and may be proposed candidate genes associated with this disease since a previous study found that oncogenic mutations of a related gene, the *PIK3CA* which is the catalytic subunit p110 of class I phosphatidylinositol 3-kinase (*PI3K*), occur in epidermal nevi and seborrheic keratosis [20].

Among the top gene predictions the *SOS2* and the *GRB2* are involved in gingival enlargement and the *PLCB3* in epilepsy. Previous studies have revealed the relationship and affinity between *SOS2*, *SOS1* and *GRB2*, being strong candidates for gingival fibromatosis (gingival enlargement) [21, 22]. In the case of *PLCB3*, discordant results have been reported when the gene is knocked out in mice producing both embryonic lethality and normal development in different studies [23, 24]; however, it has been found that the knockout of a related gene, the *PLCB1*, developed epilepsy in mice [25].

Another set of top rated genes for neoplasms were the *MAPK1* and *MAPK3*, which were linked to several neoplasms. The MAPK pathway has long been studied as an attractive pathway for anticancer therapies. The relationship of the RAS–mitogen activated protein kinase (MAPK) signalling pathway in cancer is an area of intense research since a highly-activated MAPK pathway has been reported in many types of cancers with several inhibitors being currently under investigation for their potential application as oncology drugs [26–28].

Among the newly proposed genes as candidates to be involved in different cancer types we can mention *GNAI1*, *APC2*, *PDGFA*, *CREB3*, *PRKACA*, *CREB5*, *ATF4*, *ITPR3* and *ADCY1* among others. The whole table with all the disease-gene pairs with *p*-values at the 0.001 level or below is given as Additional file 1.

With our method, from a total of 348,882 disease-gene pairs evaluated the method produced 31,066 pairs with *p*-values at the 0.001 level or below enabling the proposal of several candidate genes as potential phenotype-causing mutation genes. Our method is able to capture the bidirectional activity of the genes in the diseases allowing finding genes with greater biological relevance.

## Conclusions

Several attempts have been made in order to classify diseases, but since these classifications have been done based on their phenotype, this approach only poorly show the real level of inclusion or interaction between different diseases. The current tendency of disease classification is based on observational correlations and existing knowledge of clinical syndromes giving more importance to the phenotypes than to the molecular interconnection between diseases resulting in poor specificity in defining diseases [2]. The present study shows that the level of interaction between diseases can in some cases be irrelevant to disease classification and individual analysis should be made in order to obtain valid results for gene and metabolic pathway interactions. This information could be potentially important for studies of enrichment or gene set analysis, since diseases should be analysed individually or re-defined under different cluster classifications in order to guarantee similar phenotype or metabolic mechanism. It was expected that diseases belonging to the same cluster would have common underlying mechanisms, including gene and metabolic pathways interactions; however, this was not the general case when the analysis was made using directionality of inclusion which shows that even when diseases from the same category tend to share several genes or metabolic pathways, the level of inclusion of this genes and pathways can vary individually and independently with respect to the disease classification.

It has been previously found that the level of comorbidity is higher between diseases sharing genes and metabolic links between them [8, 29, 30]. However, these works are based only on the number of genes or pathways shared between diseases with no account of the directionality of the inclusion in their relationship. One of the main findings in the present work is that for two diseases sharing a certain number of genes, the level of inclusion can be different between both diseases due to the different pool of genes and metabolic pathways involved in each disease, and that in most of the cases, this relationship is independent on the disease categories. This information captures the structure of diseases associations under a simple but different point of view that could be relevant to provide insights into the occurrence of disease comorbidity, with potentially important consequences for disease prevention, diagnosis and treatment.

### Limitations and future work

Disease association analysis captures only a small contribution to the observed disease co-occurrence pattern, which can strongly depend on environment, lifestyle, treatment and disease complexity. More research should be done including the involvement of different omics data in order to obtain a more detailed analysis of the influence of different factors during disease development, making it a more dynamic analysis of human conditions.

Another main finding in the present study is the creation of a network-enrichment methodology based on the standard hypergeometric method that allows the prediction of new gene-diseases pairs based on their shared metabolic pathways. The results of our enrichment analysis can be useful as guidelines in order to obtain a priori biological knowledge for future gene-disease associations in drug discovery and therapeutic applications. However, as mentioned before, it is essential to personalize the medicine research in order to understand how patient characteristics such as age and coexisting diseases affects the detection, treatment, and outcome of the different human conditions. Among the proposals for future developments based on the present study, the addition of clinical data, microarray expression data and other omics data should be included in order to capture a more dynamic disease analysis leading to a personalized level of medicine research.

## Methods

### Network analysis

An updated Morbid Map at the time of the study was downloaded from OMIM database (http://www.omim.org). Pathways (ko), modules (M) and diseases (H) were downloaded from the KEGG pathway database (http://www.genome.jp/kegg/pathway.html). The complete HGNC database can be downloaded from genenames.org (http://www.genenames.org/cgi-bin/statistics). ICD-10 codes are available on the World Health Organization (http://www.who.int/classifications/icd/en/). Network analysis and KEGG associations were performed using R (http://www.r-project.org/) and Cytoscape using edge-weighted spring embedded layout (http://www.cytoscape.org/).

### Level of inclusion

The level of inclusion between diseases was calculated following the equation:

1 $$ {\displaystyle {\tau}_{x\to y}}=\frac{{\displaystyle {n}_x}\cap {\displaystyle {n}_y}}{{\displaystyle {n}_x}} $$

where *τ* is the inclusion index (0 ≤ *τ* ≤ 1), *n*_*x*_ the number of genes or pathways in disease *X* and *n*_*y*_ the number of genes or pathways in disease *Y*.

### Network-enrichment method

The probability of finding significant gene-disease associations by random chance was calculated using the hypergeometric distribution showed below:

2 $$ p=\frac{\left(\begin{array}{c}\hfill a+b\hfill \\ {}\hfill a\hfill \end{array}\right)\left(\begin{array}{c}\hfill c+d\hfill \\ {}\hfill c\hfill \end{array}\right)}{\left(\begin{array}{c}\hfill n\hfill \\ {}\hfill a+c\hfill \end{array}\right)} $$

where *a* is the number of pathways shared between the gene and the disease, *b* is the number of pathways in the gene that are not in the disease, *c* is the number of pathways in the disease, *d* is the total number of pathways linked to diseases that are not present in the particular disease and *n* is the total number of pathways in the study. This probability was used to determine which gene-disease pairs were enriched and to determine their relative ranks. The *p*-values were calculated using Python with a matrix calculated from Table 2 as follows:

**Table 2:** Fisher’s test statistics table

	Pathways in gene	Total pathways
Pathways in disease	*a* (N_g_∩N_d_)	*c* (N_d_)
Pathways not in disease	*b* (N_g_-N_d_)	*d* (N-N_d_)

The 2x2 table used to calculate the Fisher’s test statistics, where *N*_*g*_ is the number of pathways in the gene, *N*_*d*_ the number of pathways in the disease and *N* the total number of pathways linked to diseases

3 $$ fisher\_ exact\left(\left[\left[a,b\right],\left[c,d\right]\right]\right) $$

Fisher’s test was performed using Python (scipy.stats.fisher_exact).

## Additional file

Additional file 1:
**Top rated gene-disease pairs.** Table listing all the disease-gene pairs with *p*-values at the 0.001 level or below.
